# A blood tumor marker combination assay produces high sensitivity and specificity for cancer according to the natural history

**DOI:** 10.1002/cam4.1275

**Published:** 2018-02-21

**Authors:** Tsuneo Kobayashi

**Affiliations:** ^1^ International Cancer Detection and Prevention Center Chiba City Mihama‐ku Takasu 3‐21‐1 Japan

**Keywords:** Associated tumor marker, growth‐related tumor marker, risk assessment of cancer by tumor marker combination assay, serum protein fractionation by biochemical biopsy, specific tumor marker

## Abstract

Diagnosis using a specific tumor marker is difficult because the sensitivity of this detection method is under 20%. Herein, a tumor marker combination assay, combining growth‐related tumor marker and associated tumor marker (Cancer, 73(7), 1994), was employed. This double‐blind tumor marker combination assay (TMCA) showed 87.5% sensitivity as the results, but a low specificity, ranging from 30 to 76%. To overcome this low specificity, we exploited complex markers, a multivariate analysis and serum fractionation by biochemical biopsy. Thus, in this study, a combination of new techniques was used to re‐evaluate these serum samples. Three serum panels, containing 90, 120, and 97 samples were obtained from the Mayo Clinic. The final results showed 80‐90% sensitivity, 84‐85% specificity, and 83‐88% accuracy. We demonstrated a notable tumor marker combination assay with high accuracy. This TMCA should be applicable for primary cancer detection and recurrence prevention.

## Introduction

We have reported the risk assessment utilizing tumor marker combination assay according to the natural history of cancer 1. Three serum panels comprising 90, 120, and 97 serum tubes with no information on patient age, sex, disease or races were received from the Mayo Clinic (USA), under dry‐iced conditions. Within 1 month of the initial analysis, the data were sent to Dr Roger Aamodt, Dr Corle, and David Pee of the US National Cancer Institute (NCI) data center to compare these results with the clinical diagnostic data obtained from the Mayo Clinic under double blind conditions. Dr Roger Aamodt reported a sensitivity of 87.5%, but low specificity, ranging from 30 to 76% for the analyzed data. Three months later, another clinical diagnosis from the Mayo Clinic was sent to our clinic, and we further examined these data using computer analysis. We detected the following interactions between complex tumor markers: CEA x TPA, ferritin (FT)/serum iron(Fe), CEA x TPA/FT/Fe, alpha‐1‐globulin fraction(alpha‐1) x (alpha‐2‐glubulin: alpha‐2), and (alpha‐1) x TPA 2, 3. This technique elevated both the sensitivity and specificity of the detection method, and included complex tumor marker (Fe/sialic acid (SA) to discriminate early cancer from benign disease. In addition, we used multivariate analysis to discriminate cancer from noncancer in a retrospective computer analysis of 200 Japanese cancer patients and 200 healthy residents. Furthermore, this analysis formula was applied to prospectively re‐examine the original panel of American serum samples obtained from the Mayo Clinic.

**Figure 1:**
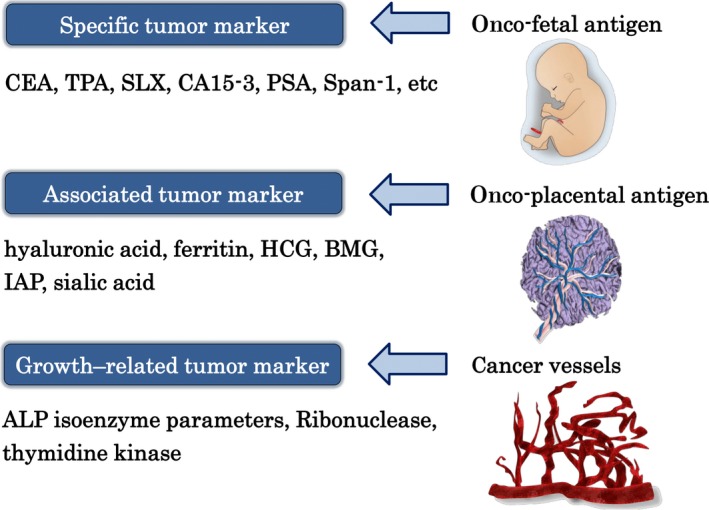
Cancer tissue actually comprises three components: tumor specific tumor marker (onco‐fetal antigen=s‐TM), tumor associated tumor marker (onco‐placental antigen=a‐TM), and growth‐related tumor marker (g‐TM) which is the information of the cancer blood vessels, as the fetus is similarly composed of the fetus, the placenta, and the chorion.

A typical cancer tissue is composed of cancer cells, interstitial tissue, and cancer vessels which looks similar to a fetus as shown in Figure 1.

For the clinical diagnostic criteria, we exploited the risk classification method utilizing serum fractionation by biochemical biopsy according to cancer progression into four cancer stages based on electrophoresis of serum protein fractionation, LDH value, and quantity of specific tumor marker level. After exploiting these new techniques, we prospectively re‐investigated the serum panels received from the Mayo Clinic to determine the sensitivity, specificity, and accuracy of detection for all 307 serum samples.

## Materials and Methods

### Materials

First serum panel (A): early lung cancer (20), early colon cancer (20), benign lung disease (15), benign colon disease (15), and healthy residents (20).

Second serum panel (B): early colon cancer (40), benign colon disease (30), and healthy residents (50).

Third serum panel(C): twenty types of early organ cancers in a total of 97 test tubes, including mammary cancer(37), prostate cancer(11), laryngeal cancer(6), urinary vesicle cancer(6), renal cancer(5), testicular cancer(5), cervical cancer(3), esophageal cancer(3), dermal cancer(3), parotid cancer(2), laryngeal cancer(2), gastric cancer(2), tongue cancer(2), nasopharyngeal cancer(2), endometrial cancer(2), mouth cavity cancer (2), uterine cervical cancer(1), lung cancer(1),penis cancer(1), and gall bladder cancer(1).

All clinical diagnostic confirmation was obtained from the Mayo Clinic.

In the third serum panel C, all samples were obtained from early stage cancers; thus, 70 serum samples from healthy residents were taken from panel A and B in order to be able to test for the sensitivity, specificity and accuracy of cancer detection in panel C. Therefore, a total of 167 samples were included in the serum panel C.

### Methods

#### Evaluation of early cancer

1. We characterized a serum sample as cancerous when two specific tumor markers(s‐TM) were positive and the alpha‐1‐globulin fraction was higher than 2.5–2.7%.
2. We characterized a serum sample as cancerous when one s‐TM was positive, more than two associated tumor markers (a‐TM) were positive, all growth–related markers(g‐TM) were positive, the alpha‐1‐globulin fraction (alpha‐1) was higher than 2.7%, and the albumin fraction was less than 63%. We discounted the contribution of inflammation from the albumin and alpha‐1‐globulin fractions according to the measure of C‐reactive protein (CRP). When serum iron (Fe)/sialic acid was higher than 0.5, we discounted the inflammation effect based on the measure of C‐reactive protein (CRP).
3. Multivariate analysis 3.

The following formula were retrospectively obtained from the data of 200 Japanese cancer patients and 200 healthy residents and prospectively applied to analyze the American serum samples. We have generated multivariate analysis formula by utilizing discrimination software program (micro‐CDA exploited by Haga in Tokyo University of Science)

1. *Z* = 125APA + 0.6ALP1 – ALP2/3‐ 17.5 This formula is derived from only alkaline phosphatase (ALP) isoenzyme parameters (sensitivity: 60%, accuracy: 66%).
2. *Z*_1_^ ^= 1.499x∛Z+2.030 × log RNase –9.617This formula is derived from g‐TM only (sensitivity: 85.5%, accuracy: 83.8%).
3. *Z*_2_ = 0.95 × log (FT)‐0.566xlog (FT/Fe)‐0.571 × log (TPAxCEA+0.01)‐1.249 × log (ALP2/3)‐0.272. This formula is derived from g‐TM and s‐TM, and its distribution is described below (Fig. 2). This formula can discriminate early cancer from noncancer regardless, of whether the multivariate analysis produces a positive or negative result.

**Figure 2:**
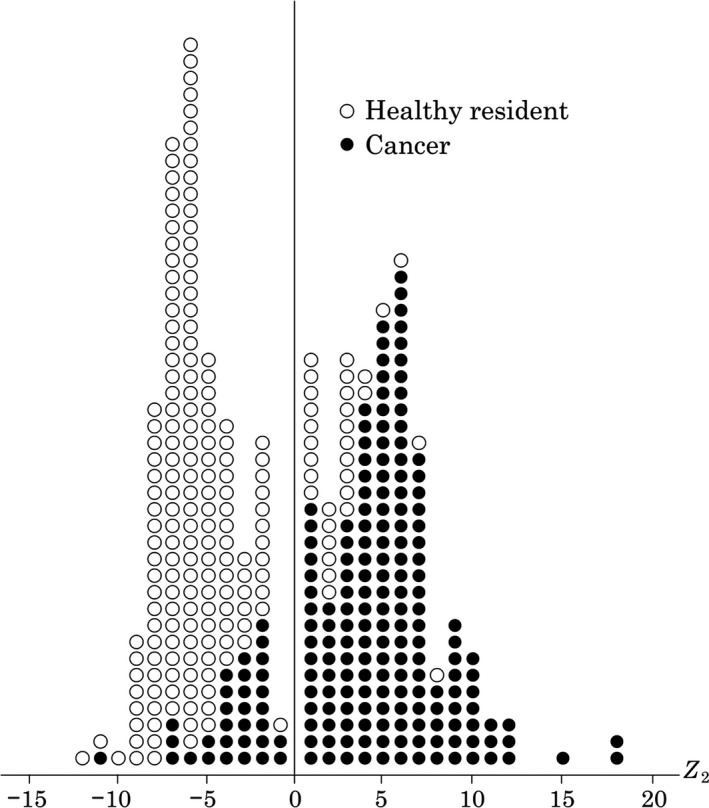
Multivariate analysis formula is derived from g‐TM and s‐TM. I will show you its distribution among 200 Japanese cancer patients and 200 healthy residents. *Z*2= 0.95 x log (FT)‐0.566xlog (FT/ Fe)‐0.571 x log (TPAxCEA+0.01)‐1.249 x log (ALP2/3)‐0.272 We have generated multivariate analysis formula by utilizing discrimination software program (micro CDA exploited by Haga in Tokyo College of Science).
4. When all growth‐related tumor markers (g‐TM) were positive and the alpha‐1‐globulin fraction were higher than 2.9%, the samples were characterized as cancerous.
5. The tumor markers were, respectively, assayed as s‐TM, a‐TM, and g‐TM.

The following specific tumor markers (s‐TMs) were examined:

Carcinoembryonic antigen: Carcinoembryonic antigen (CEA) was assayed by enzyme immunoassay using a CEA‐EIA kit(Hoffman‐Roche Co., Basel, Switzerland).

Heat‐stable alkaline phosphatase 4, 5. Heat‐stable alkaline phosphatase (HSAP) has been identified in tumor tissue and in the serum of patient with various cancers. HSAP measurements were performed according to the method of Maslow et al., with some modifications. First, 50 *μ*L of serum was heat‐treated at 65°C for 7 min, followed by treatment with a fluorescent substrate (Naphthol AS‐MX phosphate) for 20 min at 37°C subsequently, the sample was de‐proteinized with acetone, and the intensity of the fluorescence in the supernatant was measured using a fluorescence spectrophotometer (Hitachi 650‐10, Tokyo, Japan). Heat stable alkaline phosphatase (HSP) is specific fraction of ALP isoenzyme and is measured after the heat treatment at 65°C for 7 min. Of course, normal ALP is measured at 37°C.

Carbohydrate antigen 19‐9. 6: Carbohydrate antigen(CA)19‐9 was discovered by Koprowski et al. The measurement of this marker was performed by radioimmunoassay using an ELSA CA19‐9 TM RIA kit (Green Gross Co, Osaka Japan)

Tissue polypeptide antigen. 7: Tissue polypeptide antigen (TPA), a general tumor marker, was measured by radioimmunoassay using a TPA (^125^I) RIA kit (Daiichi Radio Isotope Laboratories, Ltd., Tokyo, Japan).

The following associated tumor markers (a‐TMs) were examined.

Ferritin: Ferritin was assayed by radioimmunoassay using a RIA‐Gnost Ferritin (Hoechst Behringswerke Aktion Geselshaft, Marburg, Germany). From clinical experience, the ratio of FT divided by serum iron (FT/Fe) was used as one of useful s‐TM.

Immunosuppressive acidic protein: Immunosuppressive acidic protein (IAP) is characterized by inhibiting both phytohemagglutinin induced lymphocyte blast formation and the mixed lymphocyte reaction in vitro. IAP was measured using single radial immunodiffusion with an IAP plate kit (Sanko Junyaku Co., Ltd., Tokyo, Japan).

Five microliters of sample was added to each well of an agarose gel containing anti‐IAP serum, and after incubation for 48 h at 37°C—the diameter of the preparation ring was measured.

Sialic acid: 8 High levels of sialic acid were assayed using a Hitachi 705 autoanalyzer (Hitachi, Tokyo, Japan) and the Sialic Acid Reagent kit (KT Sial Rate 50, Kyokuto Pharmaceutical Industrial, Co. Ltd., Tokyo, Japan). The determination was based on the enzyme assay according to Comb and Roseman.

Beta‐2micro‐globulin (BMG) was assayed using Latex aggregation immune turbidity 9.

Human chorionic gonadotropin (HCG) is assayed using an enzyme immunoassay according to Hussa 10.

The following growth‐related tumor markers (g‐TMs) were examined. Ribonuclease: 11, 12. The abnormal elevation of serum ribonuclease (RNase) occurs in patients with various cancers. Although this elevation is also observed in the presence of severe renal insufficiency, a creatinine test excludes this likelihood. RNase activity was determined using polycytidylic acid (Yamasa Co., Ltd., Choshi, Japan) as a substrate according to the method of Reddi and Holland.

As the molecular homology of angiogenin has same homology with Ribonuclease in 37%, so this RNase activity may have some connection with g‐TM 13.

ALP isoenzyme 5, 14. Alkaline phosphatase (ALP) isoenzyme was separated using cellulose acetate membrane (Taitan III kit, Helena Ltd. Tokyo, Japan) electrophoresis. We previously demonstrated that the serum ALP isoenzyme levels show significant variation depending on the condition of the disease in patients with cancer. Three parameters (ALP1, ALP2/3, and alkaline phosphatase isozyme angle=APA) were then calculated from the densitometric patterns of the ALP isoenzyme, as previously described 14, 15.

#### Thymidine kinase (TK) 16

TK activity correlates with the proliferative activity of a malignant tumor.

TK activity is typically measured using radio enzyme assay (REA) and experimental ELISA methods, which limits the clinical use of this biomarker, although recent studies in dogs with malignant lymphoma(ML) have the wide potential of this marker.

Serum protein fractionation using a biochemical biopsy technique 17.

With the progression of cancer, the fractions of serum protein altered. The albumin/globulin ratio gradually decreases, particularly, the albumin fraction decreases, and the alpha‐1‐globulin (*α*1), alpha‐2‐globulin(*α*2), and gamma‐globulin(*γ*‐g) fractions increase. Indeed, this protein fractionation change is also observed during inflammation. Thus, it is necessary to discriminate these changes according the degree of CRP, which discounts the distribution from inflammation.

As standard criteria, in stage I cancer, the albumin ratio is under 65–63%, the *α*1‐globulin fraction is 2.5–2.7%, and the *γ*‐globulin fraction is greater than 17–19%.

In stage II cancer, the albumin ratio is 60–63%, the *α*1‐globulin fraction is typically within 2.7–3% and the *γ*‐globulin fraction is greater than 19–21%.

## Results

A valuable growth‐related tumor marker was initially detected. The presence of growth‐related tumor markers was examined among 96 cancer patients with neoplasms that were morphologically progressing or regressing. Table 1 shows that ALP isoenzyme parameters, including ALP2/3 (which means the area ratio of ALP isoenzyme two divided by the area of ALP isoenzyme 3) and APA (ALP isoenzyme angle), presented good correlations. 15.

**Table 1:** Correlation between morphological changes in cancer and 4 ALP parameters

Parameters	Condition	
	Morphologically aggravated (*n* = 26)	Morphologically improved (*n* = 20)
ALP1	16/26 (62%)	11/20 (55)
ALP 2/3	25/26 (96)	17/20 (85)
APA	21/23 (91)	15/18 (83)
Ribonuclease	11/12 (92)	9/15 (60)

From these morphological data, ALP2/3 and Ribonuclease are evaluated to be useful for growth‐related tumor marker. Object study is different in Table 1 and 2.

Next, we detected appropriate growth‐related tumor markers when alpha‐immuno‐regulatory proteins (*α*1, *α*2) were increasing or decreasing Table 2.

**Table 2:** Correlation between the increase or decrease in *α*1‐ and *α*2‐globulin fractions

Parameters	Condition	
	Increase *α*1: from less to more 3.3% level, or *α*2: from less to more, 10% level	Decrease *α*1: from above to below 3.3% level, or *α*2: from above to below 10% level
ALP 1	42/86 (49%)	31/77 (40%)
ALP 2/3	78/86 (91)	66/77 (86)
APA	54/84 (64)	57/75 (76)
RNase	60/64 (94)	53/60 (88)

From these study for serum fraction changing, ALP2/3 and RNase are evaluated as good g‐TM.

These data suggest that ALP2/3 and ribonuclease (RNase) are good growth‐related tumor markers.

**Figure 3:**
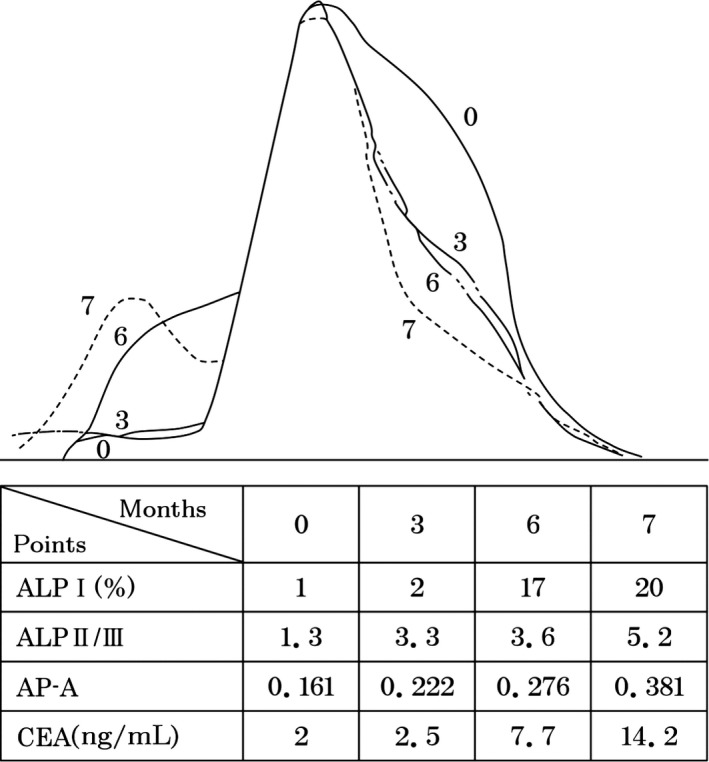
Each ALP isoenzyme parameter change according to the progression of esophageal cancer. Number in the figure are month, according to the proceeding of the month, all the parameters (ALP1, ALPII/III, and AP‐A) are progressive just like the progression of CEA value (2‐14.2). Each ALP isoenzyme parameter in patients with stage IV esophageal cancer (T3N2,P1 H2) with metastasis to the liver and lung was investigated. The patient received radiation plus local hyperthermia without surgical operation. The ALP isoenzyme parameters and CEA levels worsened with time (month) (Fig. 3). This method of ALP analysis is applicable only within normal range.

Finally, each ALP isoenzyme parameter in patients with stage IV esophageal cancer (T3N2,P1 H2) with metastasis to the liver and lung was investigated. The patients received radiation plus local hyperthermia without surgical operation. The ALP isoenzyme parameters and CEA levels worsened with time (Fig. 3). This method of ALP isoenzyme analysis system is applicable only within normal range of total ALP activity.

1. The meaning of complex marker of (Fe/SA) (Fig. 4) The ratio of serum iron divided by sialic acid (Fe/SA) is a good complex marker to discriminate early cancer from benign disease. Thus, other complex tumor markers such as TPA x CEA, FT/Fe, and TPA x CEA/FT/Fe, were examined to discriminate early cancer from healthy residents.

**Figure 4:**
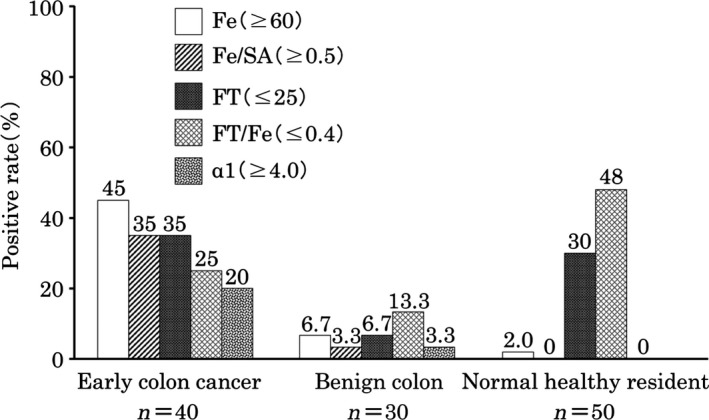
The meaning of complex marker of serum iron/ sialic acid (Fe/ SA). The ratio of serum iron divided by sialic acid (Fe / SA) is a good complex marker to discriminate early cancer from benign disease. Thus, other complex markers such as TPA x CEA, FT/ Fe, and TPA x CEA/ FT/ Fe were measured to discriminate early cancer from normal residents.
2. Complex tumor markers were also exploited to increase both the sensitivity and specificity of detection. The following table shows the data obtained for the serum samples from the Mayo Clinic (Fig. 3); Table 3.Utilizing complex tumor markers (CEA x TPA, FT/Fe, TPA x CEA/FT/Fe) increased both the sensitivity and specificity of detection. Complex tumor markers and serum protein fractionation by biochemical biopsy, were applied to examine the Mayo Clinic serum samples, including panels A to C (Table 4).
  **Table 3:** Utilizing complex tumor markers increased both the sensitivity and specificity of detection

Tumor marker	Sensitivity (%)	Specificity (%)	Accuracy (%)
(1) Mayo Clinic.^a^			
CEA (≧ 4.4 ng/ml)	17.5 (14/80)	98.5 (128/130)	67.9 (142/210)
TPA (≧125 U/L)	37.5 (30/80)	83.1 (108/130)	65.7 (138/210)
FT/Fe (≦ 0.4)	27.5 (22/80)	69.2 (90/130)	53.3 (112/210)
TPA x CEA (≧380)	28.8 (23/80)	99.2 (129/130)	72.4 (152/210)
TPA x CEA/(FT/Fe) (≧600)	31.3 (25/80)	91.5 (119/130)	68.6 (144/210)
TPA x CEA (≧380) and/or TPA x CEA/(FT/Fe) (≧600)	42.5 (34/80)	90.8 (118/130)	72.4 (152/210)

a The number of cases; colon cancer (early stage), 60; lung cancer (early stage), 20; benign colon, 45; benign lung, 15; normal, 70.

Complex tumor markers were also exploited to increase both sensitivity and specificity of detection. The upper table shows the data obtained for serum samples from Mayo Clinic. Utilizing complex tumor marker (TPA x CEA, FT/Fe, TPA x CEA/FT/Fe) increased both sensitivity and specificity.
  **Table 4:** The results of the final tumor marker combination assay for the three serum panels

	Specificity (%)	Accuracy (%)
Serum panel A: sensitivity (%)		
Early lung cancer 85(17/20)		
Early colon cancer 90(18/20)		
Benign and healthy residents	84 (42/50)	85.6 (77/90)
Serum panel B: sensitivity (%)		
Early colon cancer. 80(32/40)		
Benign and healthy residents	85 (68/80)	83.3 (100/120)
Serum panel C: sensitivity (%)		
Various early cancers: 90.7(88/97)		
Benign and healthy residents	84.3 (59/70)	88.0 (147/167)

Complex tumor markers and serum fractionation by biochemical biopsy were applied to examine the Mayo Clinic serum samples, including panel A to C.
3. The results of the final tumor marker combination assay for the three serum panels (Table 4).

## Conclusion

We can overcome high sensitivity but low specificity in the initial examination utilizing exploited various complex tumor marker, multivariate analysis formula and serum fractionation by biochemical biopsy. We could get more precise TMCA with high sensitivity and high specificity. This TMCA system has no problem against race wall. Cancer can be diagnosed according to natural history of cancer using dynamic tumor marker combination assay (TMCA), which is more sensitive than image diagnosis. With utilizing this method, we can avoid miss‐diagnosis of cancer risk assessment.

In 1988, we were able to achieve fairly high sensitivity for cancer detection (87.5%) but relatively low specificity (30–76%) on the blinded serum samples sent to our lab by Mayo Clinic in the USA. Since then, we have utilized various complex tumor makers, a multivariate analysis formula and serum fractionation by biochemical biopsy on the blinded serum samples sent from Mayo Clinic. We have now achieved a high sensitivity (80–90%) and high specificity (84–85%) for early cancer detection with serum using our Tumor Marker Combination Assay (TMCA). This TMCA system has similar accuracy for early cancer diagnosis in Japanese and United States residents. Cancer can now be diagnosed with greater ease and likely with even less expense using this dynamic TMCA. The TMCA also appear to be more sensitive than image diagnosis, and by utilizing this method, we can reduce the risk of misdiagnosing cancer.

## Discussion

Image diagnosis is usually performed on limited localized organs in advanced cancer cases and does not consistently distinguish between benign tumors and cancer tissue. The sensitivity of cancer imaging is also insufficient. Likewise, diagnosis using specific tumor markers shows low sensitivity and is insufficient. Thus, we improved the sensitivity to more than 80% using the dynamic evaluating method of TMCA. Our results indicate that this tumor marker combination assay can be used for early diagnosis, primary cancer prevention and recurrence prevention and checking for efficacy of treatment.

Herein, we developed a highly sensitive TMCA, examining specific, associated and growth‐related, complex tumor markers, to elevate both the sensitivity and specificity of detection, particularly, the ratio of serum iron/sialic acid (Fe/SA) is useful to discriminate early cancer from benign disease.

The concept that cancer tissue comprises onco‐fetal antigen, onco‐placental, and cancer vessels 18, 19, 20, 21, 22, is well established. The application of this dynamic TMCA may contribute to primary cancer prevention and may also reveal remnant cancer after surgery or be used to evaluate residual cancer through a comparison of data obtained before and after TMCA so as to carry out recurrence prevention. To date, primary cancer prevention and recurrence prevention have been achieved in 21000 residents.(will be reported in near future)

## Conflict of Interest

None.
